# Supplementary material for: Evaluation of pre-analytical factors affecting plasma DNA analysis
Source: Sci Rep. 2018 May 9;8:7375. doi: 10.1038/s41598-018-25810-0 (PMC5943304; doi:10.1038/s41598-018-25810-0)
Supplement: Supplementary file 1 — Supplemental Information [file 41598_2018_25810_MOESM1_ESM.pdf]

Evaluation of pre-analytical factors affecting plasma DNA analysis

Supplementary Information

Havell Markus, Tania Contente-Cuomo, Maria Farooq, Winnie S. Liang, Mitesh J. Borad, Shivan Sivakumar, Simon Gollins, Nhan L. Tran, Harshil D. Dhruv, Michael E. Berens, Alan Bryce, Aleksandar Sekulic, Antoni Ribas, Jeffrey M. Trent, Patricia M. LoRusso, Muhammed Murtaza

Supplemental Figure 1: Assessment of linear quantitative PCR performance for individual assays over a range of input concentrations

Supplemental Figure 2: Relative performance of individual assays within multiplexed ddPCR

Supplemental Figure 3: Comparison of ddPCR with DNA quantification by fluorometry

Supplemental Figure 4: Comparison of ddPCR with DNA quantification by electrophoresis

Supplemental Figure 5: Correlation of GMR between paired samples from different blood collection protocols

Supplemental Figure 6: Position of PCR primers and probes, relative to common SNPs in dbSNP v150

Supplemental Table 1: Primer sequences used in the ddPCR QC assay

Supplemental Table 2: Comparison sequencing library diversity with cfDNA input amounts measured using ddPCR

Supplemental Table 3: List of cell-free DNA extraction kits evaluated

Supplemental Table 4: ddPCR QC results for comparison of cfDNA extraction methods

Supplemental Table 5: ddPCR QC and sequencing results for comparison of blood collection tubes and conditions

Supplemental Table 6: List of clinical cohorts and sample processing protocols

Supplemental Table 7: ddPCR QC results from clinical samples

Supplemental Figure 1

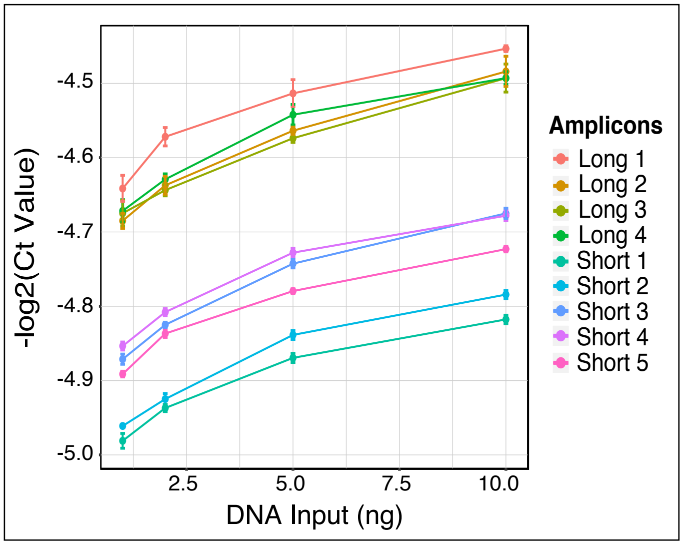

Assessment of linear quantitative PCR performance for individual assays over a range of input concentrations

To test linearity of amplification for each of the 9 loci over a range of input, we performed single-plex qPCR assays. Input DNA for this experiment was not fragmented. Each individual assay performs within expectations of linearity, with excellent correlation between cycle threshold and input DNA amounts (Pearson r between 0.935-0.981, p values$<10^{-5}$).

Supplemental Figure 2

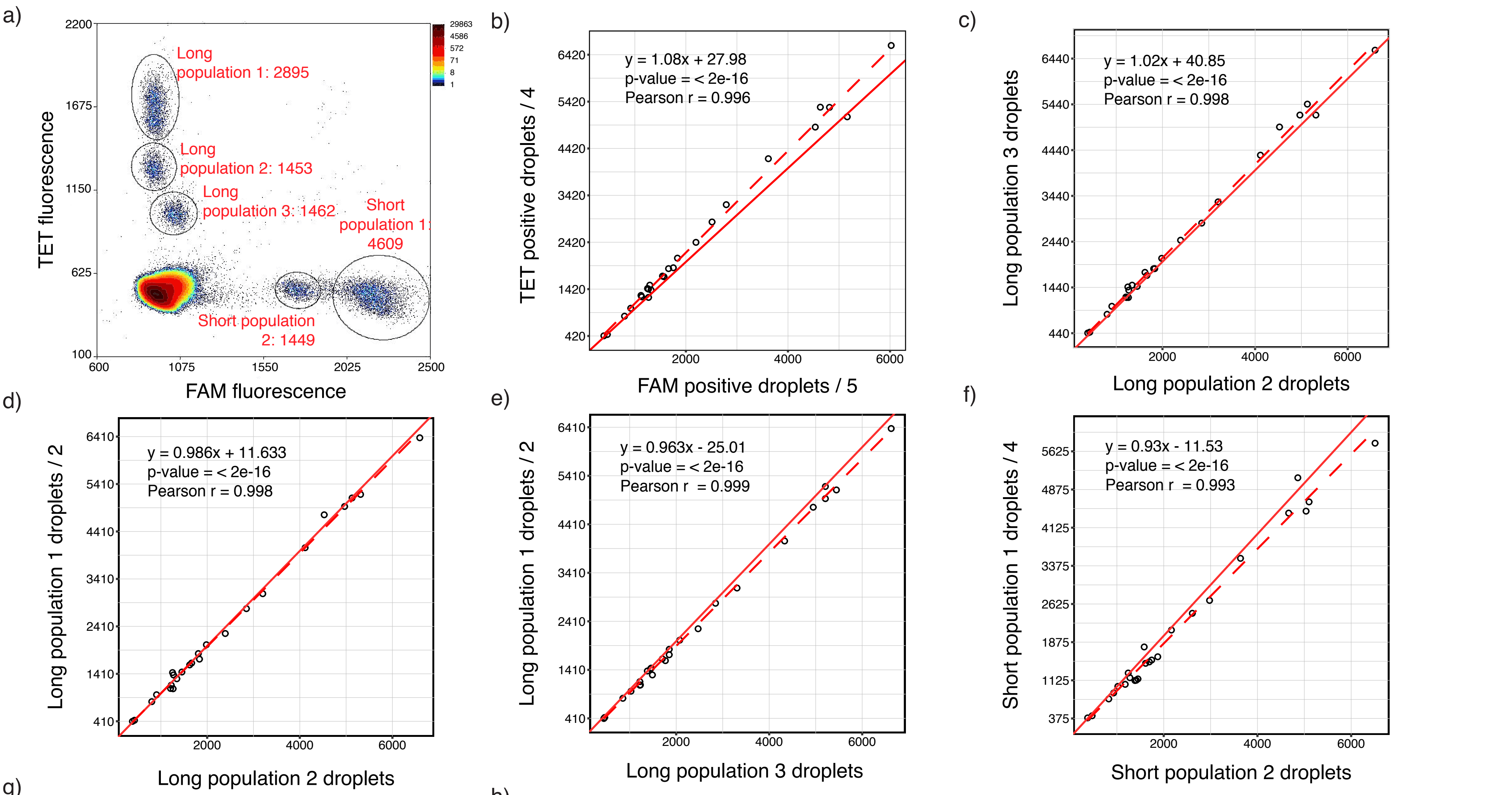

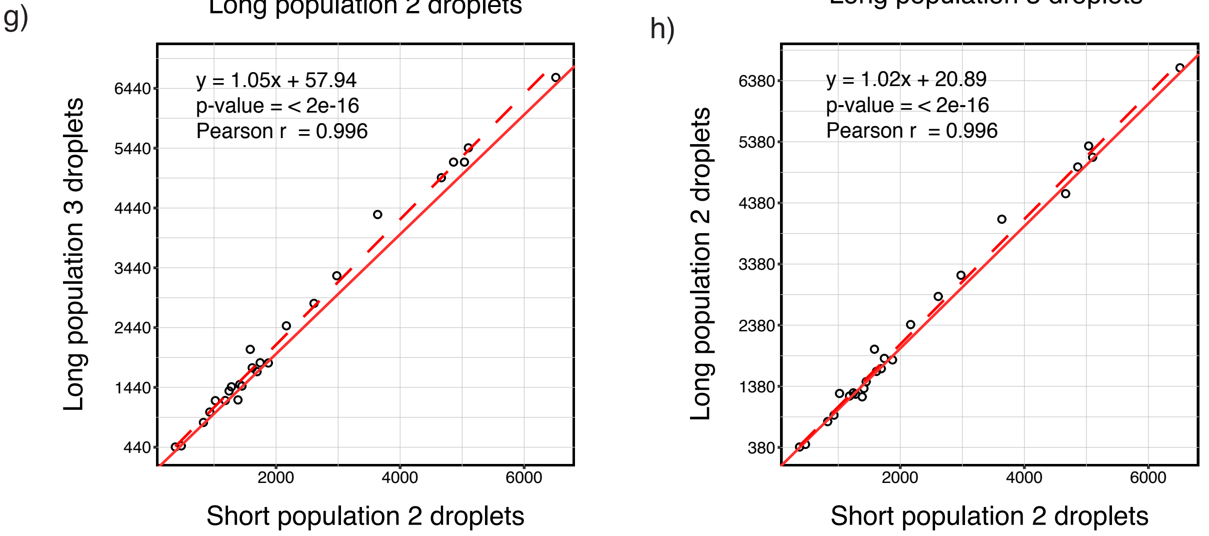

Relative performance of individual assays within multiplexed droplet digital PCR

We observed distinct droplet clusters, corresponding with differences in amplicon size and used this observation to compare performance of assays in 25 intact genomic DNA samples. (a) a representative plot of ddPCR results using our multiplexed assay. 4 long assays cluster into 3 droplet populations and 5 short assays cluster into 2 droplet populations. (b-h) comparison of performance between distinct clusters. Equivalent performance is expected since input DNA is intact. Solid red line has a slope of 1. Dotted red line is linear fit.

Supplemental Figure 3

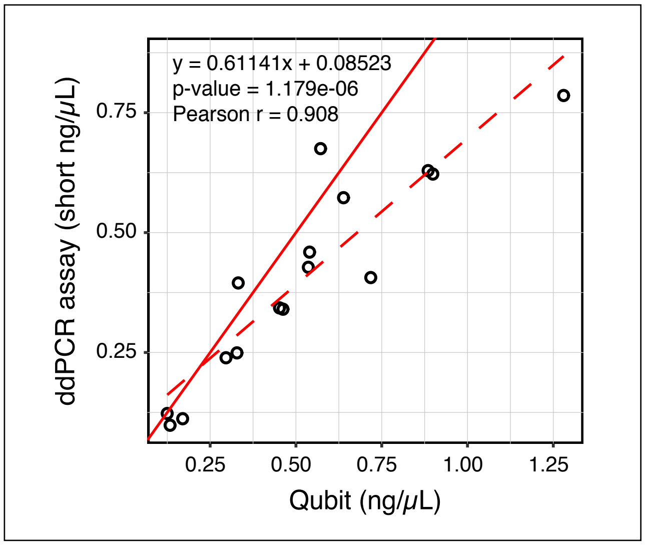

Comparison of ddPCR with DNA quantification by fluorometry

We evaluated DNA concentration in 16 samples using ddPCR and Qubit fluorometry. ddPCR concentrations are reported as average genome copies measured using the short assays (converted in ng/μL, assuming 3.3pg per haploid genome). There are two limitations in this assessment and some discrepancy is expected: 1) Qubit measures total DNA fragments regardless of their amplifiability using PCR, a feature of the ddPCR quality assessment assay because it can more accurately predict performance of downstream assays. 2) The short assays are ~70bp in size. Qubit will measure DNA fragments shorter than 70bp which ddPCR will miss. Solid line has a slope of 1. Dotted line is linear fit.

Supplemental Figure 4

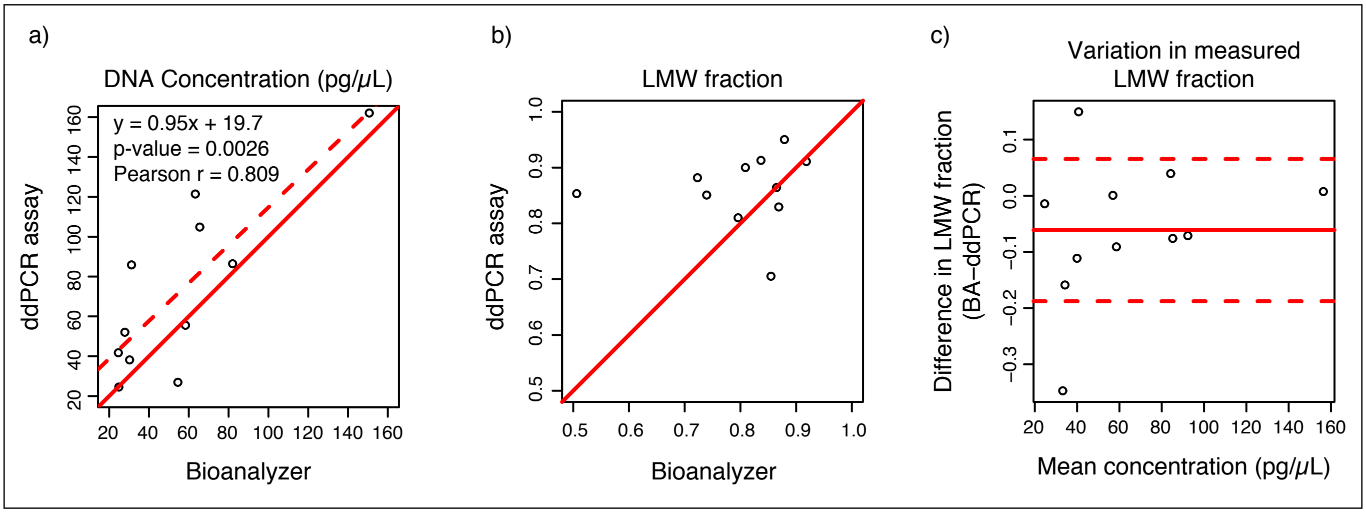

Comparison of ddPCR with DNA quantification by electrophoresis

We evaluated DNA concentration and LMW fraction in 11 samples using ddPCR and Bioanalyzer DNA High Sensitivity Assay. ddPCR concentrations are reported as low molecular weight genome copies (see text for calculation of LMW concentration), converted into pg/μL, assuming 3.3pg per haploid genome). We defined two regions in Bioanalyzer, Low Molecular Weight (LMW) from 71bp-471bp and High Molecular Weight (471bp-7000bp). In (a), we compare LMW concentration between the two methods and find strong correlation. In (b), we compare LMW fraction reported by the two methods. We calculated Bioanalyzer predicted LMW fraction: LMW/(LMW+HMW). Although there is general agreement visually between LMW fraction measured using Bioanalyzer and LMW fraction measured using ddPCR, there is no statistically significant correlation, likely due to the limited resolution of Bioanalyzer at low cfDNA concentrations. To investigate this further, we plotted the observed difference in LMW fraction in (c) against the mean concentration for each sample (mean of both BA and ddPCR measurements). We observed that the LMW fraction differences are larger for lower mean concentrations. In (a) and (b), solid line has a slope of 1. In (a), dotted line is linear fit. In (c), solid line is mean of difference in LMW fraction (mean: -0.06) and dotted lines are 1 standard deviation above and below the mean (sd:0.126).

Supplemental Figure 5

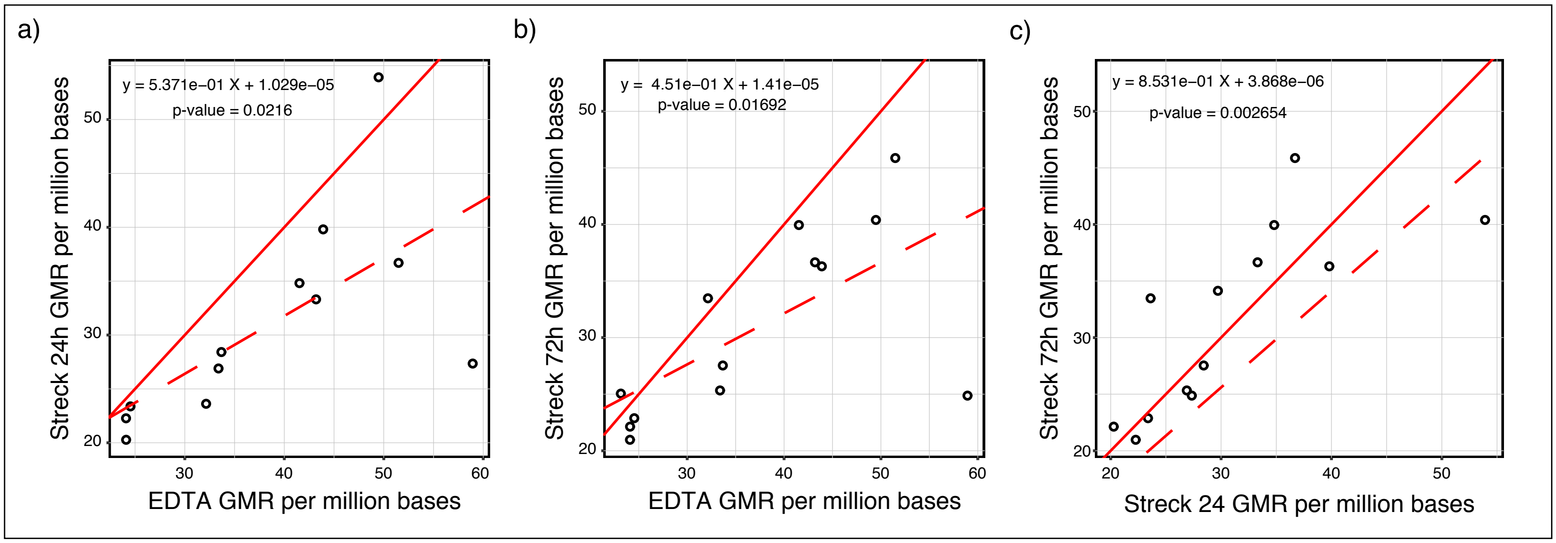

Correlation of GMR between paired samples from different blood collection protocols.

Dashed line represents linear fit as indicated. Solid line with slope of 1 included for reference. (a) Comparison of GMR between EDTA and Streck 24h showed significant correlation (Pearson r=0.652, p=0.022, n=12 pairs). (b) Comparison of GMR between EDTA and Streck 72h showed significant correlation (Pearson r=0.647, p=0.017, n=13 pairs).
(c) Comparison of GMR between Streck 24h and Streck 72h showed significant correlation (Pearson r=0.758, p=0.003, n=13 pairs).

Supplemental Figure 6

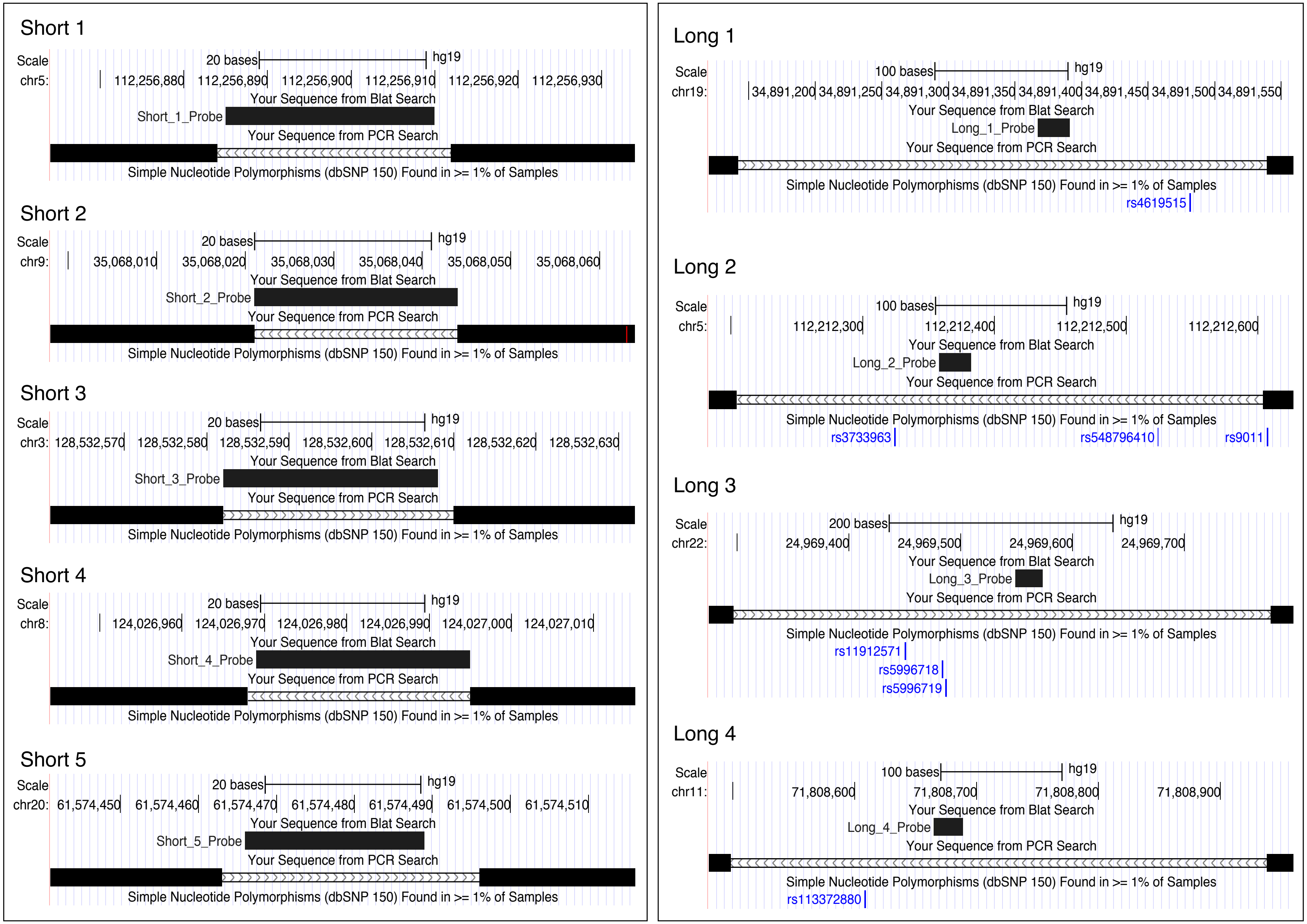

Position of PCR primers and probes, relative to common SNPs in dbSNP v150

Each panel is a snapshot from UCSC genome browser, evaluating the position of PCR primers and probes, relative to common SNPs in dbSNP v150 on hg19. All 9 probes and all except 1 of 18 primers avoid polymorphic sites. For one assay, there is a common SNP (rs9011) 4 bp upstream of the 3' position of a primer (Long 2 Forward Primer, Supplemental Table 2). However, given the position of this SNP and it is unlikely to have a significant effect on quantitative performance in ddPCR. The primer targets the reference allele which is the minor allele (minor allele fraction (p)=0.362 in dbSNP). We expect 87% samples to carry at least one copy of the non-reference allele (assuming Hardy-Weinberg equilibrium, hets(2pq)=0.462 and homozygous(q2)=0.407). Despite this, we find long 2 correlates well with other long assays (Supplemental Figure 2).
